# Supplementary material for: Ezrin Regulates the Cell Surface Localization of PD-L1 in HEC-151 Cells
Source: J Clin Med. 2022 Apr 15;11(8):2226. doi: 10.3390/jcm11082226 (PMC9030767; doi:10.3390/jcm11082226)
Supplement: Supplementary file 1 [file jcm-11-02226-s001.zip › jcm-1649630-supplementary.pdf]

Journal of
Clinical Medicine

MDPI

Supplementary Materials

Ezrin Regulates the Cell Surface Localization of PD-L1 in HEC−151 Cells

Chihiro Tanaka [1,†], Takuro Kobori [1,†], Rie Okada [1], Rina Doukuni [1], Mayuka Tameishi [1], Yoko Urashima [1], Takuya Ito [2], Nobumasa Takagaki [3], and Tokio Obata [1,*]

[1] Laboratory of Clinical Pharmaceutics, Faculty of Pharmacy, Osaka Ohtani University, Tondabayashi, 584−8540 Japan; u4117078@osaka−ohtani.ac.jp (C.T.); koboritaku@osaka−ohtani.ac.jp (T.K.); u4118025@osaka−ohtani.ac.jp (R.O.); u4118098@osaka−ohtani.ac.jp (R.D.); u4117083@osaka−ohtani.ac.jp (M.T.); urasiyo@osaka−ohtani.ac.jp (Y.U.)

[2] Laboratory of Natural Medicines, Faculty of Pharmacy, Osaka Ohtani University, Tondabayashi, 584−8540 Japan; itoutaku@osaka−ohtani.ac.jp (T.I.)

[3] Nobumasa Clinic, Kyoto, 601−8041 Japan; nobukin555@gmail.com (N.T.)

* Correspondence: obatatoki@osaka−ohtani.ac.jp (T.O.);
Tel.: +81−721−24−9371 (T.O.)

† These authors contributed equally to this work.

Original source images for immunoblots.

PD–L1

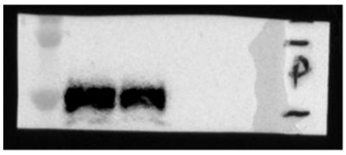

Figure 1c

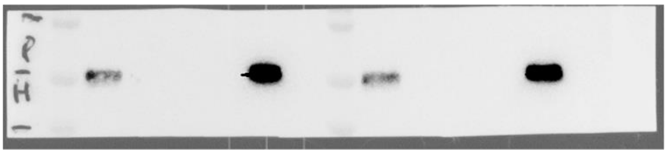

Figure 4

Ezrin

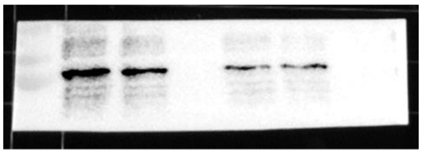

Figure 1c

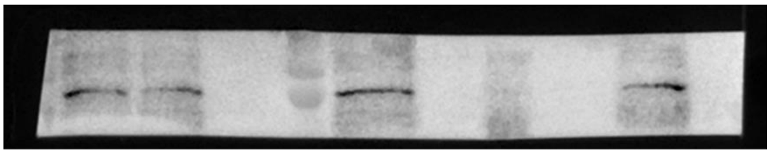

Figure 4

Radixin

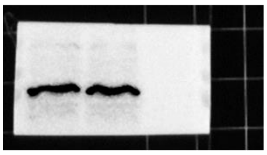

Figure 1c

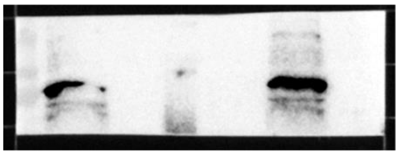

Figure 4

Moesin

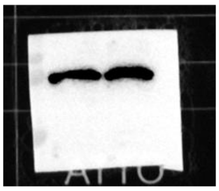

Figure 1c

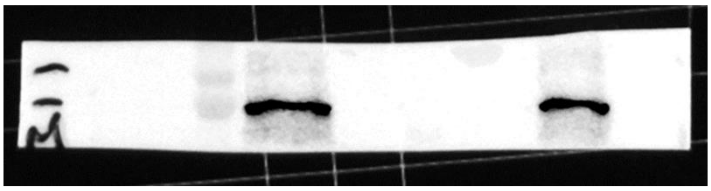

Figure 4

GAPDH

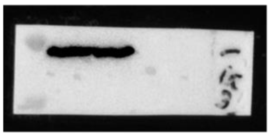

Figure 1c (PD–L1)

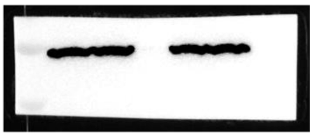

Figure 1c (Ezrin)

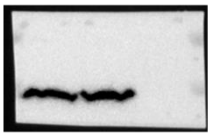

Figure 1c (Radixin)

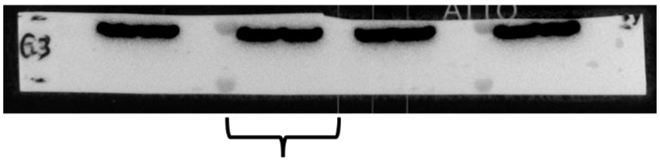

Figure 1c (Moesin)

Figure S1. Original source images for immunoblots. The original western blotting membrane to detect the protein expression of programmed death ligand−1 (PD−L1), ezrin, radixin, and moesin as well as the corresponding glyceraldehyde−3−phosphate dehydrogenase (GAPDH) used as a loading control shown in Figure 1c or Figure 4. Blots are labeled according to the corresponding Figure panel within the main manuscript.

RNA interference−mediated knockdown of target genes in HEC−151 cells.

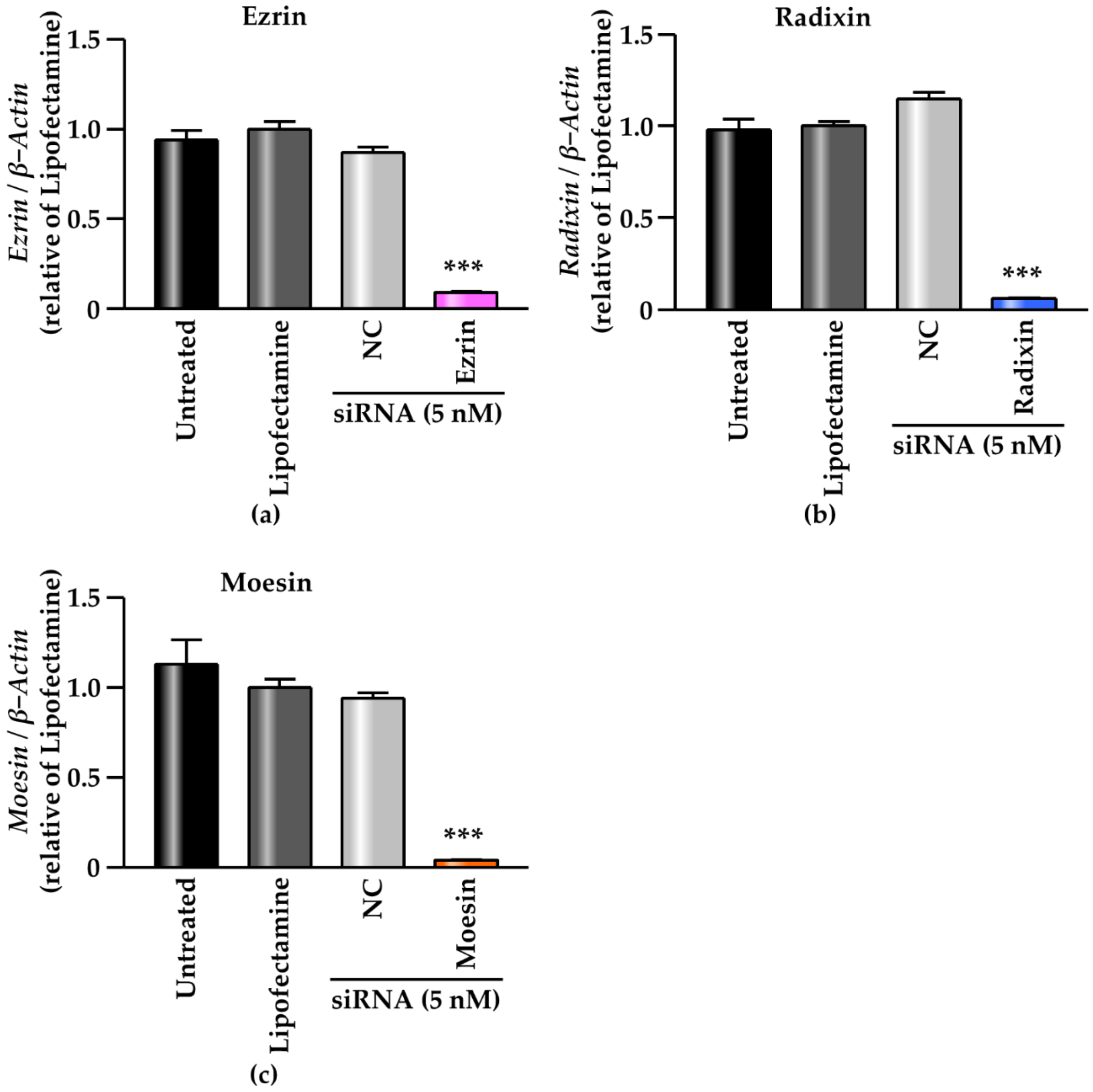

Figure S2. RNA interference−mediated knockdown of target genes in HEC−151 cells. Cells were treated with the transfection medium (Untreated), transfection reagent (Lipofectamine), nontargeting control (NC) siRNA, and specific siRNAs for ezrin, radixin, or moesin at the concentrations of 5 nM, and then cultured for 4 days. Each column shows the mRNA expression levels of (**a**) ezrin, (**b**) radixin, and (**c**) moesin normalized with β−Actin in cells from each treatment group relative to that in cells treated with Lipofectamine alone as determined by real−time reverse transcription−polymerase chain reaction. $n = 3$, ***$p < 0.001$ vs. Lipofectamine. All data were expressed as the mean ± SEM and analyzed by one−way ANOVA followed by Dunnett's test.

Table S1. List of primers used in the present study.

**Gene**	**Primer sequence (5′→3′)**
h–β–Actin (forward)	TGGCACCCAGCACAATGAA
h–β–Actin (reverse)	CTAAGTCATAGTCCGCCTAGAAGCA
h–Ezrin (forward)	ACCATGGATGCAGAGCTGGAG
h–Ezrin (reverse)	CATAGTGGAGGCCAAAGTACCACA
h–Radixin (forward)	GAATTTGCCATTCAGCCCAATA
h–Radixin (reverse)	GCCATGTAGAATAACCTTTGCTGTC
h–Moesin (forward)	CCGAATCCAAGCCGTGTGTA
h–Moesin (reverse)	GGCAAACTCCAGCTCTGCATC
h–PD–L1 (forward)	CAATGTGACCAGCACACTGAGAA
h–PD–L1 (reverse)	GGCATAATAAGATGGCTCCCAGAA

Table S2. List of antibodies used in the present study.

Antibodies	Source	Cat#	Dilution
rabbit anti−ezrin	Cell Signaling Technology	3145	1:2,000 (WB)<br>1:50 (IF)
rabbit anti−radixin	Gene Tex	GTX105408	1:2,000 (WB)<br>1:100 (IF)
rabbit anti−moesin	Cell Signaling Technology	3150	1:2,000 (WB)<br>1:50 (IF)
Alexa Fluor 488−conjugated rabbit anti−PD−L1	Cell Signaling Technology	25048	1:50 (IF)
Alexa Fluor 488−conjugated goat anti−rabbit IgG	Thermo Fisher Scientific	R37116	1:25 (IF)
Alexa Fluor 594−conjugated goat anti−rabbit IgG	Thermo Fisher Scientific	R37117	1:25 (IF)
HRP−conjugated rabbit anti−PD−L1	Cell Signaling Technology	51296s	1:1,000 (WB)
mouse anti−GAPDH	Merck	MAB374	1:20,000 (WB)
HRP−conjugated anti−rabbit IgG (heavy + light)	SeraCare Life Sciences	5220−0336	1:10,000 (WB)
HRP−conjugated anti−mouse IgG (heavy + light)	SeraCare Life Sciences	5220−0341	1:10,000 (WB)
rabbit anti−PD−L1	Cell Signaling Technology	13684	1:30 (IP)
rabbit IgG isotype control	Cell Signaling Technology	3900	1:30 (IP)
APC−conjugated mouse anti−human PD−L1	BioLegend	329708	4.0 μg/test (FC)

western blotting; WB, immunofluorescence; IF, immunoprecipitation; IP, flow cytometry; FC, horseradish peroxidase; HRP, allophycocyanin; APC
